# GMMAD: a comprehensive database of human gut microbial metabolite associations with diseases

**DOI:** 10.1186/s12864-023-09599-5

**Published:** 2023-08-24

**Authors:** Cheng-Yu Wang, Xia Kuang, Qiao-Qiao Wang, Gu-Qin Zhang, Zhen-Shun Cheng, Zi-Xin Deng, Feng-Biao Guo

**Affiliations:** 1 Department of Respiratory and Critical Care Medicine, Zhongnan Hospital of Wuhan University, Wuhan, China; 2 Key Laboratory of Combinatorial Biosynthesis and Drug Discovery, Ministry of Education and School of Pharmaceutical Sciences, Wuhan University, Wuhan, China

**Keywords:** Disease, Metabolite, Microbe, Association score, GMMAD database

## Abstract

**Background:**

The natural products, metabolites, of gut microbes are crucial effect factors on diseases. Comprehensive identification and annotation of relationships among disease, metabolites, and microbes can provide efficient and targeted solutions towards understanding the mechanism of complex disease and development of new markers and drugs.

**Results:**

We developed **G**ut **M**icrobial **M**etabolite **A**ssociation with **D**isease (GMMAD), a manually curated database of associations among human diseases, gut microbes, and metabolites of gut microbes. Here, this initial release (i) contains 3,836 disease-microbe associations and 879,263 microbe-metabolite associations, which were extracted from literatures and available resources and then experienced our manual curation; (ii) defines an association strength score and a confidence score. With these two scores, GMMAD predicted 220,690 disease-metabolite associations, where the metabolites all belong to the gut microbes. We think that the positive effective (with both scores higher than suggested thresholds) associations will help identify disease marker and understand the pathogenic mechanism from the sense of gut microbes. The negative effective associations would be taken as biomarkers and have the potential as drug candidates. Literature proofs supported our proposal with experimental consistence; (iii) provides a user-friendly web interface that allows users to browse, search, and download information on associations among diseases, metabolites, and microbes. The resource is freely available at http://guolab.whu.edu.cn/GMMAD.

**Conclusions:**

As the online-available unique resource for gut microbial metabolite-disease associations, GMMAD is helpful for researchers to explore mechanisms of disease- metabolite-microbe and screen the drug and marker candidates for different diseases.

**Supplementary Information:**

The online version contains supplementary material available at 10.1186/s12864-023-09599-5.

## Background

Our gut harbours trillions of microbes which play essential roles in many physiological and pathological processes. The disturbances of gut microbiome homeostasis can cause many diseases. For example, the enrichment of *Fusobacterium nucleatum* can induce colorectal cancer metastasis [1]. Many other diseases are also affected by microbes, such as Parkinson disease and diabetes, yet their mechanisms are still unclear [2, 3]. Thanks to advances in technologies such as sequencing, we are now able to observe and analyse the composition and status of gut microbes. This can help us to diagnose diseases early and even propose new treatments based on the increase and decrease of different microbial populations in diseases [4]. Although numerous studies uncover the mechanism among diseases, microbes, and metabolites, these associations are still scattered in the literature [5].

In recent decades, more and more studies have shown that microbes affect human health through their metabolites [6]. The metabolite is one of the key factors that drive the interaction between human gut microbes and diseases. For example, Trimethylamine N-oxide (TMAO) is a metabolite derived from the gut microbiota, which has been widely reported to be associated with cardiovascular disease [7]. Recently, some studies also reported that TMAO might be a key activator of antitumor immunity [8, 9]. Another metabolite associated with cardiovascular disease is phenylacetylglutamine (PAGln). Stanley L. Hazen et al. found gut microbes that converted dietary phenylalanine into phenylacetic acid. Then, they combined with Gln and generated the PAGln. PAGln could enhance platelet activation and be associated with adverse cardiovascular events such as myocardial infarction and stroke [10]. Based on these findings, the metabolites are believed to have the potential to act as drugs of various disease, and the disruption of metabolites also identified as signatures of many diseases.

Some resources focused on the association of disease-microbe or microbe-metabolite. For example, HMDAD collect text-mining-based microbe-disease associations from peer-reviewed publications [11]. The current version contains 483 disease-microbe entries, which include 39 diseases and 292 microbes. The gutMDisorder is a main repository for dysbiosis of the gut microbiota in disorders and interventions, [12] which contains 2,263 associations between 579 gut microbes and 123 disorders or 77 interventions in Human. The mBodyMap is a curated database for tissue microbes’ associations with diseases and health [13], which contains a total of 63,148 runs, including 14,401 metagenomes and 48,747 amplicons related to health and 56 human diseases. Some researchers also developed some predicting methods to dig the potential disease-microbe associations. NTSHMDA can predict the human microbe-disease association based on random walk by integrating network topological similarity [14]. ABHMDA is a model to reveal the associations between diseases and microbes by calculating the relation probability of a disease-microbe pair using a strong classifier, which can be applied to new disease without any known related microbes [15]. Many other methods and models can predict disease-microbe associations [16–19]. On the other hand, there are a few resources that store the metabolites of microbes, such as VMH [20] and NJS16 [21]. However, there is still a shortage of resources of diseases’ associations with metabolites of gut microbes.

**Fig. 1:**
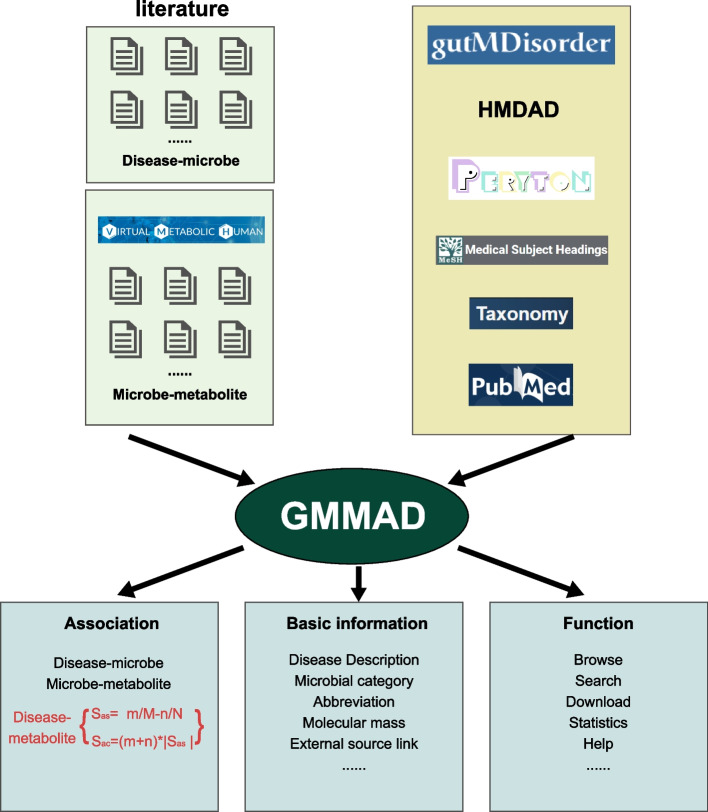
The process of data collection and database construction. Upper part shows the process of the data collection, including manually curating literature and integrating public databases. The left panel describes our manual extraction of experimental microbe-disease information. The right panel gives the list of public databases of information source. Lower part shows the types of associations, basic information of each association entry and GMMAD functions. The left panel shows the three types of associations. Disease-metabolite is the core association, and it is provided exclusively by our database. The middle panel tells the basic information of each entry, including details of disease description, microbial category, molecular mass of metabolite and so on. The right panel provides a list of GMMAD’s main functions

To fill this gap, we constructed the Gut Microbial Metabolite Association with Disease (GMMAD), which aims to provide users comprehensive metabolism related information of gut microbes in human diseases. Users can easily browse associations among diseases, microbes, and metabolites stored in GMMAD, and screen the potential candidate disease signatures or drugs from our predicted disease-metabolite associations (Fig. 1). The current version of GMMAD documents the 45,058 meaningful disease-metabolite associations which are predicted and involved with 31 diseases and 2,299 metabolites. We expect GMMAD to be a timely and valuable resource for gut microbes’ research.

## Materials and methods

### Data collection for the first two associations

All the names of diseases in GMMAD followed the principle from the Medical Subject Heading disease categories [22], and it contained 113 diseases. The annotation information of microbes was collected from the Taxonomy database [23]. All the metabolites’ details were obtained from the PubChem database [24]. For the association data, we integrated several public databases. The disease-microbe associations were collected from HMDAD, gutMDisorder, and Peryton [25]. All data from other public databases should meet the naming standards of disease (MeSH), microbe (ncbi Taxonomy) and metabolite (PubChem). Because most of the current sequencing technologies for microbes, such as metagenomic sequencing and 16sRNA sequencing, can accurately identify only at the genus level, the microbes in our disease-microbe associations are all defined on the genus level. The microbe-metabolite associations were obtained from VMH [20], NJS16 [21], and the study of Han S. et al. [6]. The microbes collected from VMH were in the strain level. We map the genus level of gut microbes for disease-microbe association to the strain level of metabolite-microbe association based on lineage data obtained from Taxonomy. In detail, if microbes were found to increase\decrease under the genus level in a particular disease, then it is assumed that all strains included in that genus have increased/decreased at the disease status. For example, if *Faecalibacterium* (in genus level) is decreased in obesity, it is assumed that the *Faecalibacterium cf. prausnitzii KLE1255* and *Faecalibacterium prausnitzii A2-165* (two strains of microbes belonging to *Faecalibacterium*) were both decreased in obesity. Furthermore, we used the keywords combination to search related literatures in PubMed, such as ‘gut microbiota AND cancer AND metabolites’, ‘intestinal microbiota AND diabetes AND metabolites’, etc. Then, we downloaded all published literature and available supplementary files describing the associations among diseases, microbes, and metabolites. Finally, we manually extracted experimentally supported associations from selected articles by at least two researchers. For all the association, we removed the contradictory entries to ensure that the data in GMMAD were harmonious. The redundant entries stored in the GMMAD database were removed (upper part of Fig. 1). Values of the above two association are discrete with the first one having two directions and the latter one being “appearing” or “deficiency”. For the latter one, we only record those metabolites appearing in one specific microbe.

### Association strength score and confidence score

For the core disease-metabolite relationships, we defined one quantitative value. We supposed that common metabolites in multiple decreased numbers were beneficial to treat specific human disease, while the ones in multiple increased microbes would promote the development of that disease [26, 27]. We defined an association strength score to infer the association between the diseases and metabolites of gut microbes. We defined the number of microbes which increase/decrease in a specific disease as M/N, and the number of the increasing and decreasing microbes which generated a specific metabolite as m/n. The formula for the association strength score is:

$${S}_{as}= \frac{m}{M}-\frac{n}{N}$$

Meanwhile, we defined a confidence score to judge the credibility of association strength scores:

$${S}_{ac}=\left(m+n\right)*\left|{S}_{as}\right|$$

Because the microbial levels in the two associations were inconsistent (disease-microbe with genus level and microbe-metabolite with strain level), we used the affiliation data of microbial lineage (finally, microbe in strain level) to establish the association. ${S}_{\mathrm{as}}$ of three and five diseases are all 1 and -1, respectively. After checking, it was found that only one microbe associated with these diseases, such as ‘Colitis, Microscopic’ and ‘Gastroesophageal Reflux’. ${S}_{\mathrm{as}}$ simulated the abundant alternation of a specific metabolite by counting the increasing or decreasing number of microbial strains in a disease sample compared to a normal sample’s gut. If a metabolite appears in only decreased microbes, its association score ${S}_{ac}$ will have a very large negative value and in principle, it will be beneficial for the treatment of the disease or at least strongly associated with the disease. Hence, a strong positive correlation score indicated that the metabolite might be considered as a potential marker of disease, while a strong negative correlation score indicated that the metabolite might be a candidate drug for disease or potential diagnostic marker. We only considered those with the absolute value of association strength score > 0.05 and confidence score > 1 to be meaningful.

### Database construction

Finally, all data in GMMAD are stored and managed using MySQL (version 5.6.50). The web interfaces were built in HTML/CSS/JS on Linux and Apache platform. The data processing programs are written in Python (version 2.7.5). The GMMAD has been tested in the Google Chrome (version 104.0.5112.81) and Firefox (version 101.0.1) browsers. The GMMAD database is freely available at http://guolab.whu.edu.cn/GMMAD.

## Results

### Database content

**Fig. 2:**
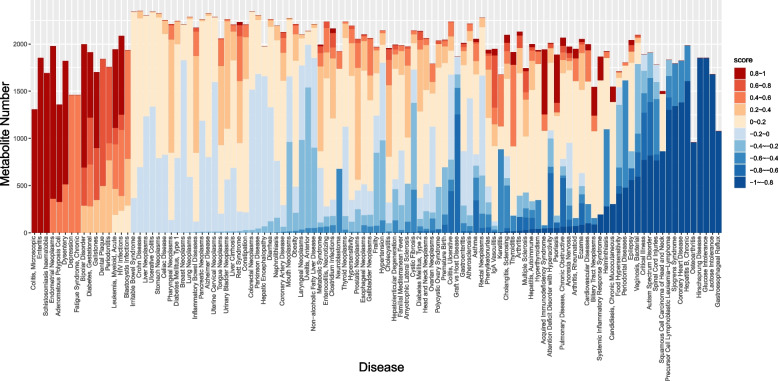
Distribution of association strength scores in 113 different diseases. Diseases were shown in the deceasing order of the mean *S*_*as*_ of associated metabolites

**Fig. 3:**
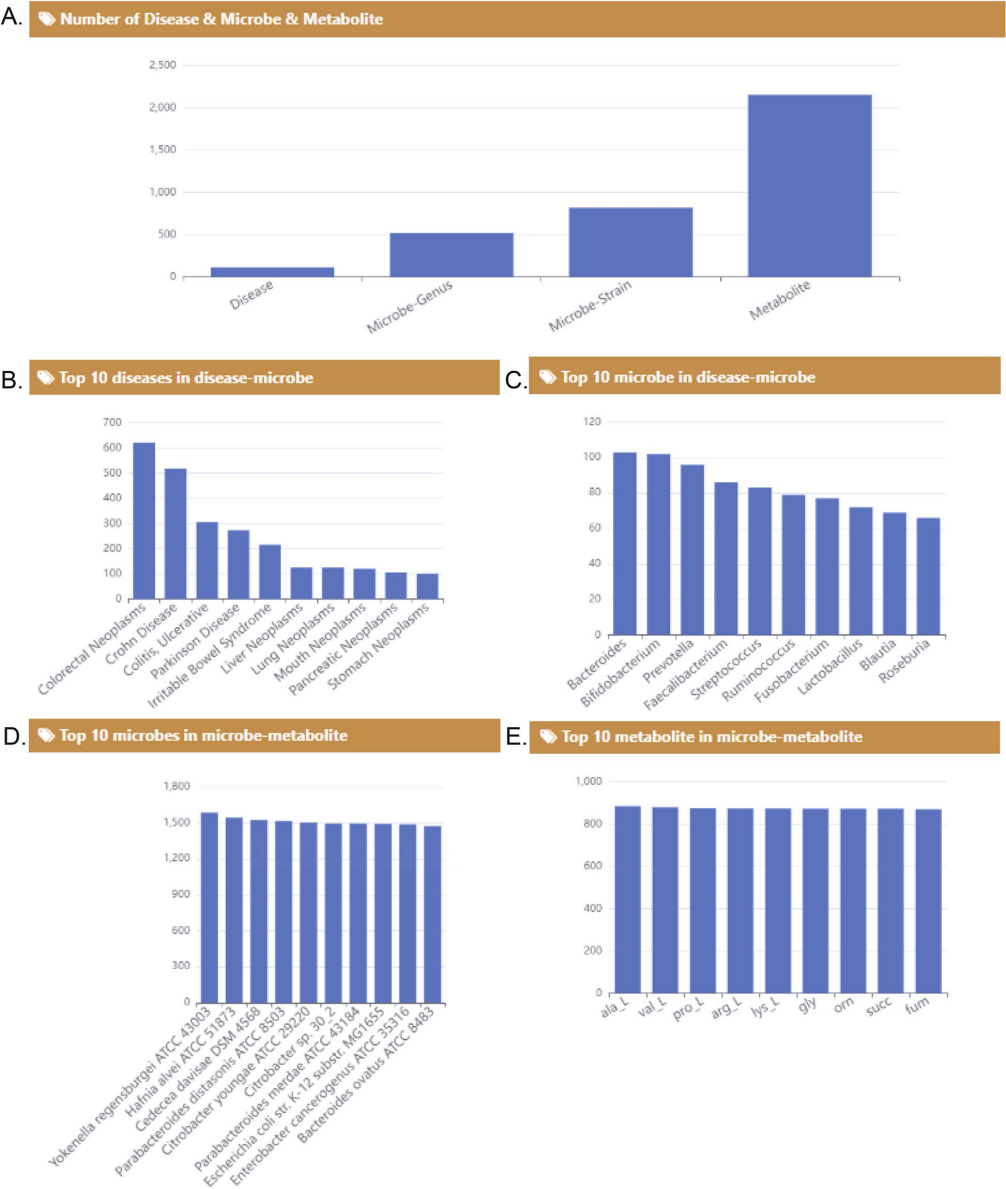
Statistic of data in GMMAD. **A** The number of diseases, microbes and metabolites in GMMAD. **B** The top 10 diseases in disease-microbe associations. **C** The top 10 microbes in disease-microbe associations. **D** The top 10 microbes in microbe-metabolite associations. **E** The top 10 metabolite s in microbe-metabolite associations

With our method, we predicted 220,690 entries of disease-metabolite associations, the confidence scores are distributed from -1 to 1 (Fig. 2). Integrating and compiling all the associations we obtained from public databases and papers, GMMAD contains 3,836 disease-microbe associations and 879,263 microbe-metabolite associations which involve 113 diseases, 893 gut microbes, and 2,448 metabolites of gut microbes (Fig. 3A). Finally, there are 45,058 meaningful disease-metabolite associations by scoring prediction, which involve 31 diseases and 2,299 metabolites. Since each microbe produces thousands of metabolites and each disease has hundreds of associated microbes, the number of associations for the three elements disease-microbe-metabolite is huge. Showing all these associations is not helpful for user search, and it is hard for database maintenance. Thus, GMMAD only shows associations between any two of the three terms: disease, microbe and metabolite. For each entry, GMMAD restored the human disease name (e.g. Crohn disease), microbe name (e.g. *Faecalibacterium*), metabolite name (e.g. Actinomyces georgiae DSM 6843), alteration pattern (e.g. increase and decrease), the experimental method (e.g. 16S rRNA sequences), alteration strength score and confidence score of disease-metabolite associations, disease detail (e.g. disease description), metabolite information (e.g. charged formula, average molecular mass), and microbe information. The ID of disease, microbe, metabolite and PMID were also provided which could be linked to MeSH, Taxonomy, PubChem and PubMed for more details.

Figure 3B-E show the top 10 diseases, microbes, and metabolites in the different types of associations. The colorectal neoplasm is the disease with the most entries (Fig. 3B). This suggested that colorectal neoplasm is closely connected to gut microbes and their metabolites. *Bacteroides* are the microbe which associated with the most diseases (Fig. 3C). In addition, *Yokenella regensburgei ATCC 43003* and ala_L (L-Alanine) are the microbe and metabolite with the most entries in microbe-metabolite associations (Fig. 3D, E)*.*

### GMMAD implementation

**Fig. 4:**
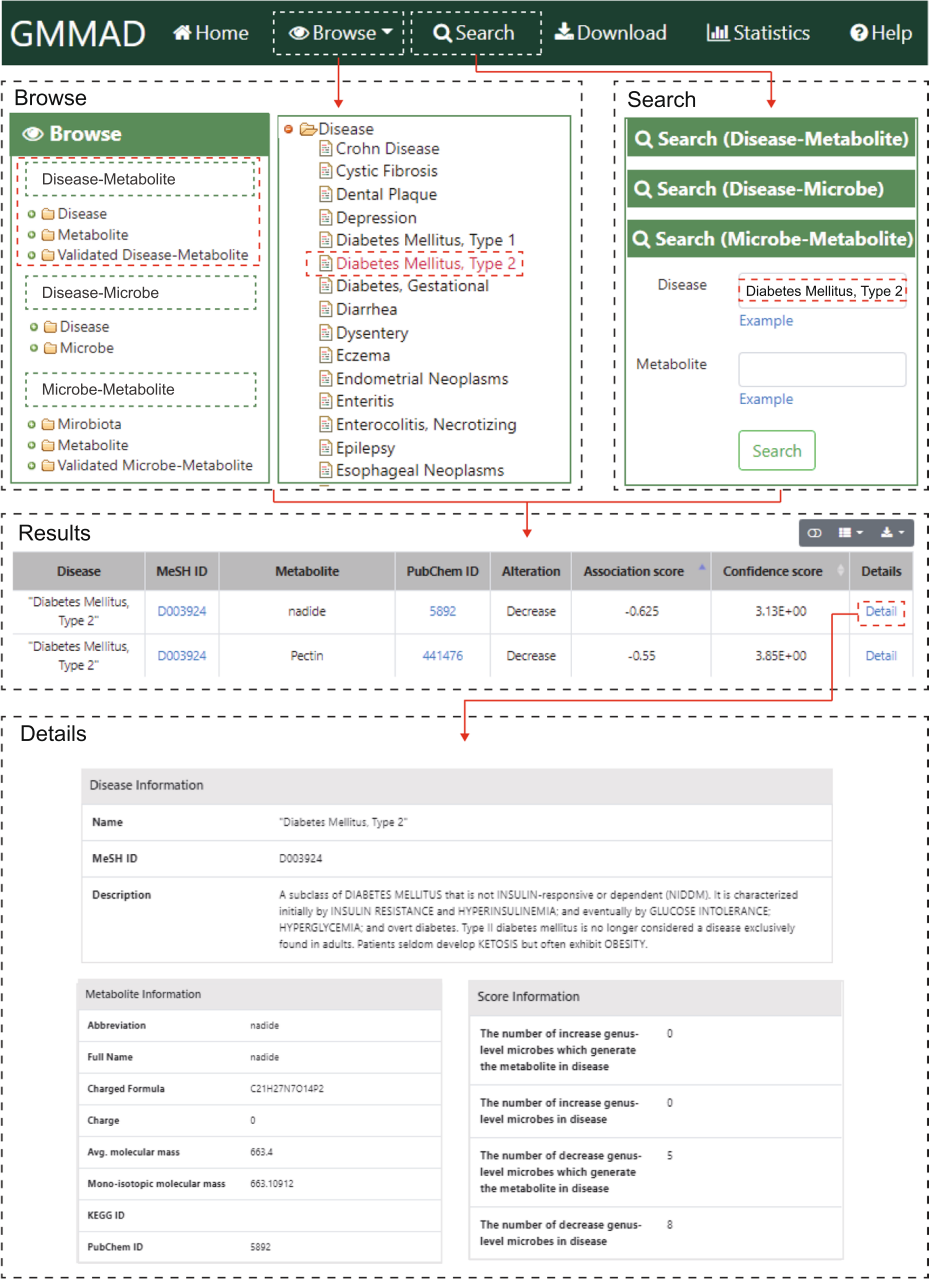
Schematic workflow of GMMAD. Browse part: User can browse all three types of association data in GMMAD. Search part: User can search the interested association by typing the key word. Results part: All the results will be shown in the table which includes information about each term. Details part: User can click ‘Detail’ to obtain more details about disease, microbe, and metabolite

The GMMAD provides a user-friendly interface that allows users to browse, search, and download (lower part of Fig. 1). The tree browser organizes the data according to three types of associations: “Disease-Metabolite”, “Disease-Microbe” and “Microbe-Metabolite”. Each type of root category is divided into several sub-categories named “Disease”, “Metabolite”, “Microbe” and “Validated associations” (Fig. 4). By clicking one term in these sub-categories, all associations belonging to the corresponding term would be listed on the right table. On the ‘Search’ page, users can search associations according to name and ID of diseases, microbes, and metabolites (Fig. 4). Example names for searching are provided when users click the “Example” on the bottom of each search box. In the results table, users can click the icon in the upper right corner to download the search results. The ‘Detail’ link of each entry leads to the more detailed information including the microbes’ family, description of diseases, formula of metabolites, samples information and so on (Fig. 4). The ‘Download’ page allows users to download all information on disease-metabolite, disease-microbe and microbe-metabolite associations data in.txt format. GMMAD also provides external links to the related public databases. All tutorials and details are shown in the ‘Help’ page.

### Validation of predicted disease-metabolite association

To prioritize and filter the meaning of disease-metabolite association and reveal the most interesting results, we defined an association strength score and a confidence score. As a rough evaluation of the accuracy of our method, we got 138 experimental disease-metabolite association from 49 published studies. Among them, 36 associations have meaning scores and 26 (72.2%) could be given consistent direction of disease-metabolite association (Supplementary table 1). We do not aim to pick out all the disease-metabolites associations, and our main purpose is to make our chosen association genuine in high probability and deserved to be studied as drug or marker candidates.

For example, by querying “Diabetes Mellitus, Type 2” (T2DM) in the “Search (Disease-Metabolite)” query box, GMMAD provides the 2137 T2DM associated metabolites. Each entry has an association strength score and a confidence score, which the user can browse in order of score value. For example, we sort all the “Diabetes Mellitus, Type 2” related entries according to their association strength scores, and the “nadide” is the third strongest negative association score with the T2DM (*S*_*as*_ = -0.625 and *S*_*ac*_ = 3.13). Nadide (also called NAD + , Nicotinamide Adenine Dinucleotide) is an indispensable enzyme in the human body and an important coenzyme in the tricarboxylic acid cycle. It plays a crucial role in various biological processes such as human metabolism, stress, and cell differentiation. In patients with T2DM, NAD + synthesis is severely impaired [28, 29]. Many researchers are trying to promote the synthesis of NAD + as a treatment for patients with type 2 diabetes mellitus, and several drugs based on this mechanism have been developed [30, 31]. Similarly, the “Melatonin” is the fifth strongest negative association score with the T2DM (S_as_ = -0.500 and S_ac_ = 2.00). Melatonin is well known for its sleep-promoting effects. In recent years, studies had found that melatonin has a certain hypoglycaemic effect, and its mechanism might be related to improving insulin resistance, protecting pancreatic β-cells, and regulating the hypothalamus–pituitary–adrenal axis. These findings reveal the importance of melatonin and melatonin-related bacteria and metabolites as potential therapeutic targets for type 2 diabetes [32, 33]. All of these demonstrate that our method can effectively expose the useful disease-metabolite associations.

## Discussion

Despite a lot of research on the association between gut microbes and diseases, the mechanism of gut microbes in various diseases is still unclear. In recent years, more and more researchers have found that gut microbes affect through their metabolites in diseases [34, 35].

Currently, there are many disease-microbe databases have been built, such as HMDAD [11], gutMDisorder [12] and Peryton [25]. To the best of our knowledge, none of these databases focuses on the association data of disease and metabolites of gut microbes. However, the current version of our GMMAD contains 45,058 meaningful disease-metabolite associations.

As the resource uniquely stores the quantitative association of disease and gut microbes’ metabolites, GMMAD will suggest potential markers and drugs for specific diseases. When used as drug candidates for these metabolites, the safety of these candidates will be less concerning than usual drug candidates because they naturally exist in the human gut environment. Simultaneously, the association due to their predictive essence should be experimentally validated by the research in practical application. The role of our database is to pick out those most possible drug and biomarker candidate from the gut microbes’ metabolites and ranked them with quantitative scores. By referencing our database, the experimental scientists have the refined candidates in their hands, and they deserve to pay more attention. With the accumulation of data, the one sense is the promotion of associations between the disease and microbial strains, the other sense is that the metabolome research could provide more reliable microbe-metabolite associations. We will update the database with a higher quality list of associations between specific disease and metabolites of gut microbes. In the future, besides the content update, more features will be added to the database, such as the ability to search for broad chemical classes of compounds beyond specific compounds. Meanwhile, we will provide visualization tools for showing the associations network.

### Supplementary Information

**Additional file 1: Supplementary Table 1.** Predictive scores of experimentally validated disease-metabolite association data.

## Data Availability

GMMAD is publicly available: http://guolab.whu.edu.cn/GMMAD.
