# Supplementary material for: GMMAD: a comprehensive database of human gut microbial metabolite associations with diseases
Source: BMC Genomics. 2023 Aug 24;24:482. doi: 10.1186/s12864-023-09599-5 (PMC10464125; doi:10.1186/s12864-023-09599-5)
Supplement: Supplementary file 1 — Additional file 1: Supplementary Table 1. Predictive scores of experimentally validated disease-metabolite association data. [file 12864_2023_9599_MOESM1_ESM.docx]

**Supplementary Table 1. Predictive scores of experimentally validated disease-metabolite association data.**

| **disease** | **Taxonomy id** | **metabolite** | **PubChem id** | **type** | **pmid** | **S_as_** | **S_ac_** | **Consistent direction** |
| --- | --- | --- | --- | --- | --- | --- | --- | --- |
| Diabetes Mellitus, Type 2 | D003924 | Hippurate | 464 | Increase | 31782507 | 0.18 | 1.58 | Y |
| Diabetes Mellitus, Type 2 | D003924 | ala_L | 5950 | Increase | 31782507 | 0.13 | 1.5 | Y |
| Diabetes Mellitus, Type 2 | D003924 | akg | 51 | Increase | 31179513 | 0.13 | 1.5 | Y |
| Diabetes Mellitus, Type 2 | D003924 | alltn | 204 | Increase | 31179513 | 0.25 | 2.75 | Y |
| Diabetes Mellitus, Type 2 | D003924 | cis-Aconitate | 643757 | Increase | 31179513 | 0.25 | 2.75 | Y |
| Diabetes Mellitus, Type 2 | D003924 | Citraconate | 643798 | Increase | 31179513 | 0.25 | 2.75 | Y |
| Diabetes Mellitus, Type 2 | D003924 | urea | 1176 | Increase | 31179513 | 0.25 | 2.75 | Y |
| Diabetes Mellitus, Type 2 | D003924 | orot | 967 | Decrease | 31179513 | 0.13 | 1.5 | N |
| Diabetes Mellitus, Type 2 | D003924 | Creatine | 586 | Increase | 32295805 | 0.25 | 2.75 | Y |
| Diabetes Mellitus, Type 2 | D003924 | urate | 1175 | Decrease | 32295805 | 0.88 | 5.25 | N |
| Diabetes Mellitus, Type 2 | D003924 | xan | 1188 | Increase | 31179513 | 0.13 | 1.5 | Y |
| Diabetes Mellitus, Type 2 | D003924 | phe_L | 6140 | Increase | 33276482 | 0.13 | 1.5 | Y |
| Diabetes Mellitus, Type 2 | D003924 | tyr_L | 6057 | Increase | 33276482 | 0.13 | 1.5 | Y |
| Diabetes Mellitus, Type 2 | D003924 | leu_L | 6106 | Decrease | 29570613 | 0.13 | 1.5 | N |
| Diabetes Mellitus, Type 2 | D003924 | ile_L | 6306 | Increase | 29570613 | 0.13 | 1.5 | Y |
| Diabetes Mellitus, Type 2 | D003924 | val_L | 6287 | Increase | 29570613 | 0.13 | 1.5 | Y |
| Diabetes Mellitus, Type 2 | D003924 | succ | 1110 | Increase | 29434314 | 0.13 | 1.5 | Y |
| Non-alcoholic Fatty Liver Disease | D065626 | trypta | 1150 | Decrease | 30660825 | -0.33 | 3.31 | Y |
| Non-alcoholic Fatty Liver Disease | D065626 | nh4 | 222 | Increase | 29523305 | -0.18 | 3.88 | N |
| Non-alcoholic Fatty Liver Disease | D065626 | etoh | 702 | Increase | 33490737 | -0.24 | 4.94 | N |
| Obesity | D009765 | Trigonelline | 5570 | Decrease | 20212498 | 0.17 | 2.17 | N |
| Obesity | D009765 | xan | 1188 | Decrease | 20212498 | 0.08 | 1.58 | N |
| Obesity | D009765 | Lactic acid | 612 | Increase | 19930761 | 0.13 | 1.38 | Y |
| Obesity | D009765 | phe_L | 6140 | Increase | 19930761 | 0.08 | 1.58 | Y |
| Obesity | D009765 | isoval | 10430 | Increase | 19930761 | -0.17 | 1.17 | N |
| Obesity | D009765 | phe_L | 6140 | Increase | 28628112 | 0.08 | 1.58 | Y |
| Obesity | D009765 | tyr_L | 6057 | Increase | 28628112 | 0.08 | 1.58 | Y |
| Obesity | D009765 | leu_L | 6106 | Increase | 28628112 | 0.08 | 1.58 | Y |
| Obesity | D009765 | ile_L | 6306 | Increase | 28628112 | 0.08 | 1.58 | Y |
| Obesity | D009765 | val_L | 6287 | Increase | 28628112 | 0.08 | 1.58 | Y |
| Obesity | D009765 | glu_L | 33032 | Increase | 28628112 | 0.08 | 1.58 | Y |
| Obesity | D009765 | for | 284 | Increase | 23154580 | 0.08 | 1.58 | Y |
| Irritable Bowel Syndrome | D043183 | srtn | 5202 | Increase | 33407171 | -0.06 | 2.91 | N |
| Irritable Bowel Syndrome | D043183 | 4abut | 119 | Increase | 29852727 | 0.07 | 13.76 | Y |
| Arthritis, Rheumatoid | D001172 | urcan | 736715 | Increase | 34670873 | -1 | 2 | N |
| Arthritis, Rheumatoid | D001172 | urate | 1175 | Increase | 34670873 | 1 | 2 | Y |
